# Karyopherin Alpha 2-Expressing Pancreatic Duct Glands and Intra-Islet Ducts in Aged Diabetic C414A-Mutant-CRY1 Transgenic Mice

**DOI:** 10.1155/2019/7234549

**Published:** 2019-04-24

**Authors:** Satoshi Okano, Akira Yasui, Shin-ichiro Kanno, Kennichi Satoh, Masahiko Igarashi, Osamu Nakajima

**Affiliations:** ^1^Research Center for Molecular Genetics, Institute for Promotion of Medical Science Research, Faculty of Medicine, Yamagata University, Yamagata 990-9585, Japan; ^2^Department of Functional Genomics, Innovative Medical Science Research, Graduate School of Medical Science, Yamagata University, Yamagata 990-9585, Japan; ^3^Division of Dynamic Proteome in Cancer and Aging, Institute of Development, Aging and Cancer, Tohoku University, Sendai 980-8575, Japan; ^4^Division of Gastroenterology, Tohoku Medical and Pharmaceutical University, Sendai 983-8512, Japan; ^5^Division of Diabetes and Endocrinology, Yamagata City Hospital Saiseikan, Yamagata 990-8533, Japan

## Abstract

Our earlier studies demonstrated that cysteine414- (zinc-binding site of mCRY1-) alanine mutant mCRY1 transgenic mice (Tg mice) exhibit diabetes characterized by the reduction of *β*-cell proliferation and by *β*-cell dysfunction, presumably caused by senescence-associated secretory phenotype- (SASP-) like characters of islets. Earlier studies also showed that atypical duct-like structures in the pancreas developed age-dependently in Tg mice. Numerous reports have described that karyopherin alpha 2 (KPNA2) is highly expressed in cancers of different kinds. However, details of the expression of KPNA2 in pancreatic ductal atypia and in normal pancreatic tissues remain unclear. To assess the feature of the expression of KPNA2 in the development of the ductal atypia and islet architectures, we scrutinized the pancreas of Tg mice histopathologically. Results showed that considerable expression of KPNA2 was observed in pancreatic *β*-cells, suggesting its importance in maintaining the functions of *β*-cells. In mature stages, the level of KPNA2 expression was lower in islets of Tg mice than in wild-type controls. At 4 weeks, the expression levels of KPNA2 in islets of Tg mice were the same as those in wild-type controls. These results suggest that the reduction of KPNA2 might contribute to *β*-cell dysfunction in mature Tg mice. Additionally, the formation of mucin-producing intra-islet ducts, islet fibrosis, and massive T cell recruitment to the islet occurred in aged Tg mice. In exocrine areas, primary pancreatic intraepithelial neoplasias (PanINs) with mucinous pancreatic duct glands (PDGs) emerged in aged Tg mice. High expression of KPNA2 was observed in the ductal atypia. By contrast, KPNA2 expression in normal ducts was quite low. Thus, upregulation of KPNA2 seemed to be correlated with progression of the degree of atypia in pancreatic ductal cells. The SASP-like microenvironment inside islets might play stimulatory roles in the formation of ductal metaplasia inside islets and in islet fibrosis in Tg mice.

## 1. Introduction

Cryptochrome proteins (CRYs) play indispensable roles as key constituents in the molecular time keeping processes underlying the mammalian circadian clock [1–3]. We previously generated transgenic mice (previously labeled as CRY1-AP Tg mice [4–6] or afterward C414A CRY1 Tg mice [3]: hereinafter designated as Tg mice) ubiquitously expressing mCRY1 with a mutation by which cysteine414 (the zinc-binding site of mCRY1 [7, 8]) was replaced by alanine. In addition to unusual circadian rhythms in locomotor activities [4, 9], Tg mice showed early-onset diabetes mellitus similar to maturity-onset diabetes of the young (MODY) characterized by *β*-cell dysfunction [3–6]. An earlier report described that lowered proliferation of *β*-cells is accountable for age-dependent loss of *β*-cells in Tg mice [3, 6]. Using young Tg mice, we demonstrated that islet cells in Tg mice show unique gene expression patterns similar to those of senescence-associated secretory phenotype (SASP) [3]. We also reported that hyperplasia of the ductal cells, whose morphology is reminiscent of pancreatic intraepithelial neoplasias (PanINs), is observed more frequently according to age in the pancreas of Tg mice [3]. Actually, PanINs are known as precursor lesions of pancreatic ductal adenocarcinoma (PDAC) [10]: among the most lethal malignancies affecting humans [11]. Intraductal papillary mucinous neoplasms (IPMNs) are cystic pancreatic neoplasms that also cause PDAC [10]. Pancreatic duct glands (PDGs) were characterized recently as a new kind of ductal atypia producing gastric mucins: pouch-like structures budding from pancreatic ducts [12]. Reportedly, PDGs function as a progenitor niche for the ductal epithelium that might develop to IPMNs [13]. Also, the replication of the cells of PDGs was demonstrated to increase in type 2 diabetes mellitus in humans in association with increased PanINs [14]. The growth of PDG cells also increases in type 1 diabetes mellitus in humans [15]. Consequently, from the perspective of preventing pancreatic cancer in diabetes, the molecular characterization for PanINs/IPMNs and PDGs is important.

Karyopherin *α*2 (KPNA2), which is implicated in nucleocytoplasmic transport of various proteins [16], has been demonstrated as showing aberrantly high expression in cancers of several types [16]. Although earlier reports have described that KPNA2 is involved in the regulation of GLUT2 [17], an important glucose transporter in the pancreatic *β*-cell, our knowledge of the detailed roles of KPNA2 in the transportation machineries for the maintenance of *β*-cells remains incomplete. Malfunction of the shuttling mechanisms must be deeply associated with the dysfunction of pancreatic *β*-cells in Tg mice. Furthermore, KPNA2 is known to be involved in various phenomena such as cellular differentiation and differentiation-coupled formation of the cellular circadian clock [16, 18]. Nevertheless, the exact distribution of KPNA2 in the tissues of the pancreas, including endocrine cells in the pancreas, remains unclear. Very recently, KPNA2 expression has been reported in human IPMNs [19]. This result suggests that the KPNA2 level is a hallmark of pancreatic pre-cancerous lesions. However, KPNA2 expression has not been reported for other potential pre-cancerous lesions such as PanINs and PDGs.

To explore age-dependent changes in islet architecture and to explore the development of ductal atypia, which might be related to unique SASP-like features of islets, we scrutinized not only the islets but also exocrine areas in the pancreases with emphasis on KPNA2 expression in aged Tg mice.

## 2. Materials and Methods

### 2.1. Animals

Male Tg mice (TGs [high expression line]) and their littermates (WTs) were used for experiments as described in earlier reports [4–6]. We classified the mice used for experiments into three groups depending on age: young age (4 weeks), early middle age (4.4 months), late middle age (12–13 months), and aged (17–23 months). The pancreases of mice were harvested as described in an earlier report [5]. Mice apparently having tumors or other anomalies were excluded from experiments. In all experiments, mice were treated in accordance with the guidelines of Yamagata University.

### 2.2. Immunohistochemical Analysis and Histological Examinaion

**Figure 1:**
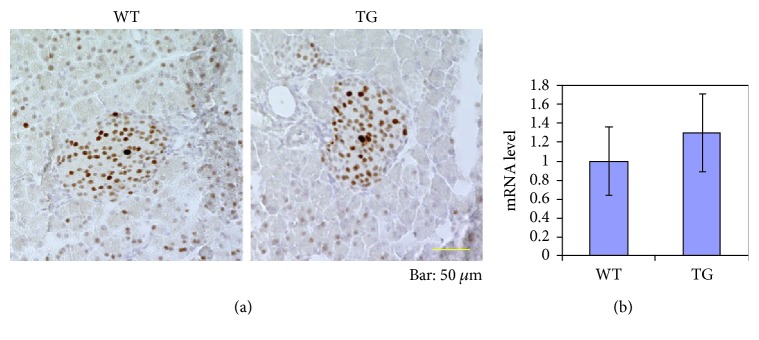
(a) Immunostaining of pancreatic islets in 4-week-old mice for KPNA2 with hematoxylin counter-staining. Pancreas sections from WTs and TGs were stained with antibodies to KPNA2. Representative images of islets are shown [WTs (left panel) and TGs (right panel)]. Bar, 50 *μ*m. No obvious difference in staining was observed between WTs and TGs at 4 weeks. (b) mRNA expression of KPNA2 in the islet. Islets were collected from TGs as well as WTs at 4 weeks of age. The relative levels of mRNA were measured by real-time PCR and normalized to the corresponding HPRT mRNA levels. In each representation, the mean value for WTs was set to 1. Data are means ± SE for 3 mice per group. No significant difference exists between WTs and TGs, (*t* test).

Immunohistochemical analyses using paraffin-embedded pancreas sections (3 *μ*m) were conducted mainly as described in earlier reports [5, 6]. Relevant primary antibodies are listed as the following: keratin 17/19 (rabbit polyclonal, D4G2; Cell Signaling Technology Inc.), KPNA2 (rabbit polyclonal, ab84440; abcam), insulin (mouse monoclonal, sc-377071; Santa Cruz Biotechnology Inc.), glucagon (mouse monoclonal, sc-57171; Santa Cruz Biotechnology Inc.), F4/80 (rabbit polyclonal, MF48000; Thermo Fisher Scientific Inc.), PDX-1 (rabbit polyclonal, KR059; TransGenic Inc.), amylase (mouse monoclonal, sc-46657; Santa Cruz Biotechnology Inc.), alpha-Smooth Muscle Actin (alpha-SMA; mouse monoclonal, 14-9760-32; Thermo Fisher Scientific Inc.), and CD3 (rabbit polyclonal, D4V8L; Cell Signaling Technology Inc.). Each antibody was diluted to an appropriate concentration in phosphate-buffered salts (PBS) containing 1% of bovine serum albumin (BSA; Sigma-Aldrich, A7906), and then the solutions were used for the treatment of pancreas sections with primary antibodies. For the experiments of negative controls, the slices were treated with the solution without primary antibodies, but otherwise the procedures for negative controls were the same as for the ordinary tests. In order to compare 4 weeks of TGs and WTs for the immunostaining of KPNA2 (for Figure 1(a)), the color development procedure was increased evenly. For mucin detection, the sections were stained with an alcian blue solution (Muto Pure Chemicals Co. Ltd.) followed by counter-staining of nuclei with nuclear fast red (ScyTek Laboratories Inc.). For detecting fibrosis, Picro-Sirius red staining was done with a kit according to the manufacturer's instructions (Picro-Sirius Red Stain Kit; ScyTek Laboratories Inc.).

### 2.3. Quantification of mRNA by Real-Time PCR

Islets were isolated from mice as described in our previous report [6]. The samples of cDNA derived from the isolated islets and the whole pancreas of mice used in the experiments are acquired as described in our previous report [6]. Real-time PCR analysis was conducted as described in the earlier report [6]. Expression levels are expressed as relative values with respect to mHprt levels, as described there. The primer sequences were as follows: KPNA2 sense, 5′-CTTCTCCGCTACAGGAAAACCGGAA-3′; KPNA2 antisense, 5′ -TTTCCGAGCAGCTTGAGTAGCTTGG-3′; Reg2 sense, 5′-CCAGGTAGCTGAAGAAGACTTCCCC-3′; Reg2 antisense, 5′-TCCCCCCAGGTCAAACGGTCTT-3′; Reg3*β* sense, 5′-ATTAGTTGCCCCAAGGGCTCCCAG-3′; Reg3*β* antisense, 5′-GAAGCCTCAGCGCTATTGAGCACAG-3′; HPRT sense, 5′-GGACCTCTCGAAGTGTTGGATACAGG-3′; HPRT antisense, 5′-CTGGCAACAT CAACAGGACTCCTCGT-3′.

### 2.4. Statistical Analysis

For each experiment, a *t*-test was used to compare the mean values. Differences between means were inferred as significant for *P* < 0.05. Data are presented as mean ± SE.

## 3. Results

### 3.1. KPNA2 Expression in Ductal Atypia

**Figure 2:**
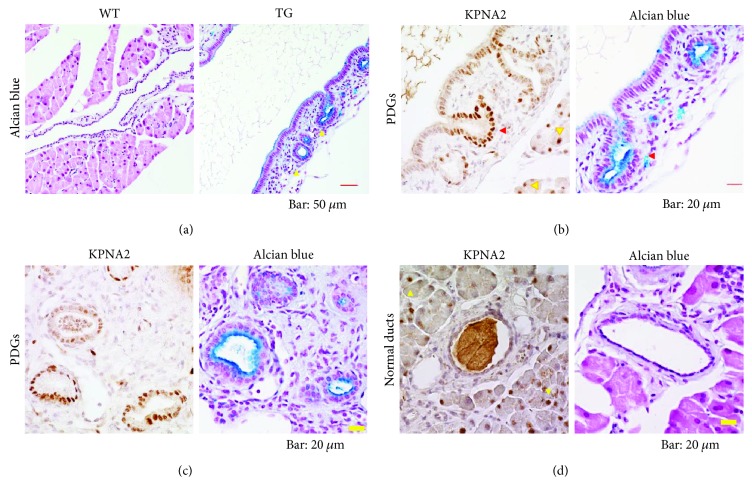
KPNA2 expression in PanIN-like ducts and PDGs in aged mice. (a) Representative images of alcian blue-stained pancreas of WT mouse (left) and TG mouse (right). The mucinous PanIN-like ducts in TG are morphologically different from ducts in WT of comparable size. In the mesenchyme around the PanIN-like duct, compartments of PDG, some of which are branched from the PanIN-like duct, are visible (yellow triangles). Bar, 50 *μ*m. (b, c, d) Left panels: immunostaining of pancreas sections of for KPNA2 (shown in brown). Right panels: alcian blue-staining, (b) representative pictures of both PanIN-like and PDG ductal structures in TG, (c) PDG compartments embedded in the mesenchyme in TG, and (d) normal ductal cells in WT. A high expression of KPNA2 in the nuclei of PanIN-like cells is discernible. KPNA2 is especially highly expressed in mucinous PDG cells ([b] red triangle). KPNA2 was observed in acinar cells too ([b and d, left panels] yellow triangle). Only quite low expression of KPNA2 was observed in the normal duct (d). Bar, 20 *μ*m.

Because the appearance and growth of the dysplastic ductal structures in TGs become prominent with age [3], we examined the pancreases obtained from 17–23-month-old mice (aged group of mice) to characterize atypical ductal dysplasia in TGs. The sections of pancreases were stained with alcian blue (staining for mucin) and subsequently with fast red (staining for nuclei) to ascertain the extent of atypia from the perspectives of the mucin production and of the morphological features of cells. In Figure 2(a) and Supplementary Figure 1 (low magnification views), the typical appearance of a main duct in WTs is shown. The ductal epithelium cells were low cuboidal and/or squamous cells surrounded by a thin layer of mesenchyme (Figure 2(a)). In TGs, ductal dysplasia having some morphological features in common with pancreatic intraepithelial neoplasias (PanIN) appeared frequently (Figure 2(a)): the pancreatic lesions consist of taller columnar cells with abundant supranuclear cytoplasm and basally located nuclei (Figures 2(a) and 2(b) and Supplementary Figure 1). It was also observed that the ductal dysplasia produced mucin (Figures 2(a) and 2(b)). The mucin production is one characteristic of the cells of pancreatic atypical ductal cells including PanIN. Based on their relative vertical lengths of supranuclear cytoplasm to their nuclei, we inferred that those atypical ductal cells correspond to immature stages of PanIN, which were still not fully grown to PanIN (hereinafter, we designate them as PanIN-like cells). The PanIN-like cells in TGs were surrounded by the thick area of the fibrotic tissue (Figure 2(a)). Results show that PDGs, which consist of low height ductal cells having highly mucinous features, appeared around the PanIN-like ductal structure in the fibrotic tissue in TGs. Reportedly, PDGs are out-pouches extended from ducts [12]. Consistent with the report, some PDGs indeed appeared as pouch-like branches that are connected directly with the PanIN-like duct (Figures 2(a) and 2(b)). As expected, in the pancreatic fibrosis area of surrounding mucin-producing atypical ductal structures in TGs, the mass of alpha-SMA positive cells was located (Supplementary Figure 2). These results suggest strongly that the activated pancreatic stellate cells play central roles in the formation of fibrosis, as described in the literature [20].

To investigate the possibility that KPNA2 is involved in the generation of ductal atypia of PanINs and PDGs, we examined ductal cells with respect to KPNA2 expression. Outside the pancreas, we found that KPNA2 is highly expressed in the epithelial cells of intestinal villi and intestinal crypt cells (Supplementary Figure 3). Therefore, we used the small intestine as a positive control for the staining of KPNA2. A close scrutiny of the KPNA2 staining in comparison with negative controls revealed that only faint staining of KPNA2 in the nucleus was observed in normal ductal cells (Figure 2(d), Supplementary Figure 4). These results indicate that the expression of KPNA2 is quite low in normal ductal cells (Figure 2(d), Supplementary Figure 4). Contrary to the results of normal ductal cells, pronounced expression of KPNA2 was observed in the PanIN-like ductal cells (Figure 2(b)). Enhanced KPNA2 expression was also observed in PDG cells (Figures 2(b) and 2(c)). These results suggest that the expression level of KPNA2 in ductal cells depends on the degree of their atypism (Figures 2(b) and 2(c)). To characterize the ductal cells further, we conducted immunohistochemical analyses for the transcription factor PDX-1. In the normal ductal cells, only weak PDX-1 expression was observed (Supplementary Figure 5). However, in the nuclei of PanIN-like and PDG cells, PDX-1 was expressed strongly, as reported previously ([12]; Supplementary Figure 5). Regarding the islets, the PDX-1-positive nuclei were fewer in TGs than in wild-type controls (Supplementary Figure 5), confirming our earlier-obtained results [6]. We also examined the expression of insulin and glucagon in the islets of aged mice. As expected, insulin-positive cells were markedly fewer in aged TGs (Supplementary Figure 6). Glucagon-positive cells were observed throughout the islet in aged TGs (Supplementary Figure 6), consistent with our earlier reported results [6].

### 3.2. KPNA2 Expression in Atypical Intra-Islet Ducts

**Figure 3:**
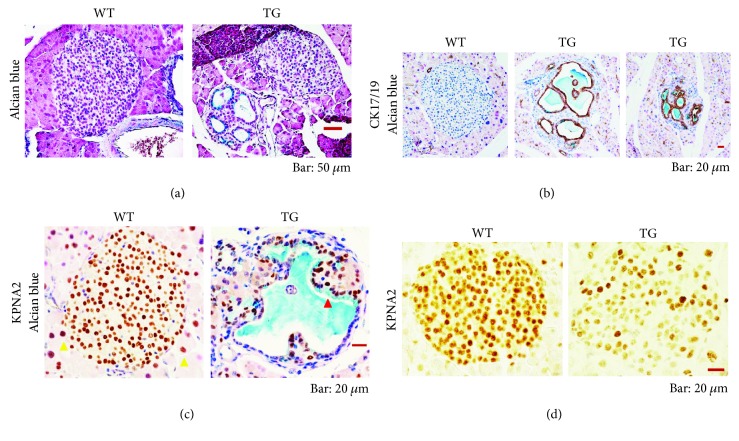
Expression of KPNA2 in pancreatic islets and intra-islet ducts in aged TGs. (a) Alcian blue-stained pancreas sections of WT (left) and TG (right). For TG, two islets are shown. The left-hand side islet contains mucin-producing intra-islet ducts. Bar, 50 *μ*m. (b) Immunostaining of pancreas sections for cytokeratin 17/19 (brown), followed by staining with alcian blue of WT (left panel) and TG (middle and right panels). Bar, 20 *μ*m. Two typical cases of islets with unusual multiple intra-islet ducts are shown. (c) Immunostaining of islets for KPNA2 (brown) with alcian blue staining of WT (left panel) and TG (right panel). Bar, 20 *μ*m. KPNA2 is located abundantly in the nuclei of endocrine cells of the islet in WT (left panel). KPNA2 resides also in the nuclei of acinar cells (left panel, yellow triangles). In the intraductal cells producing mucin of TG, a high expression of KPNA2 was observed (right panel, red triangle). (d) Immunostaining of islets for KPNA2 (shown in brown) of WT (left panel) and TG (right panel). Bar, 20 *μ*m. In TG, the expression of KPNA2 in each cell in the islet was lower than that of WT.

Furthermore, results show that unusual duct-like structures producing mucin emerged not only in the exocrine areas but also inside islets in the TGs frequently with age (Figure 3). In contrast to the PanIN-like cells, the intra-islet duct-like structures consist of cuboidal and/or squamous cells (Figure 3(a)). Among the intra-islet duct-like cells inside the lumen, small clusters of cells were often observed (Figures 3(b) and 3(c)). These results suggest strongly that the intra-islet duct-like structures contain papilla or sack-like architectures protruded toward the lumen. These intra-islet ductal cells were cytokeratin 17/19-positive (Figure 3(b)), indicating that the cells actually have ductal cell characteristics. At 4.4 months, which corresponds to the stage of early middle age, intra-islet ductal atypia was observed only slightly, even in TGs. However, during the stage of late middle age from 12 to 13 months, a large share of TGs harbored the islet with unusual ducts. All TGs had islets with intra-islet ducts in the 17–23-month group, indicating their age-dependent development. The results are presented in Table 1. Among the intra-islet cells, we observed the tendency that such cells producing large amounts of mucin show the expression of higher levels of KPNA2 as well (Figure 3(c)). These results further support the idea that the expression level of KPNA2 depends on the degree of ductal atypism. Additionally, results show that KPNA2 is highly expressed in the islet cells of WTs (Figure 3(d)). The expression of KPNA2 in the islet was markedly lower in TGs (Figure 3(d)).

### 3.3. KPNA2 Expression in Endocrine Cells in the Islet

**Figure 4:**
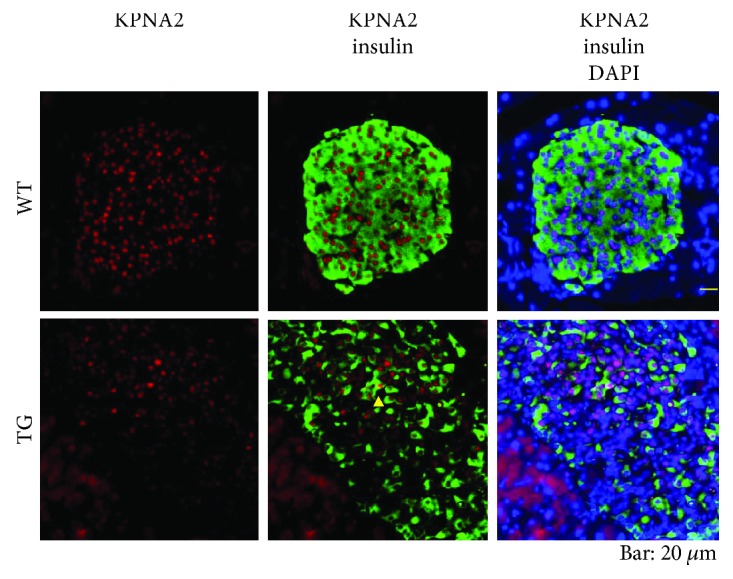
Coimmunostaining of islets for insulin and KPNA2. Pancreas sections from WTs (upper panels) and TGs (lower panels) of late middle age were costained with antibodies to insulin (green) and KPNA2 (red) and counterstained with 4,6-diamidino-2-phenylindole (DAPI; blue) for nuclear staining. Bar, 20 *μ*m. KPNA2 was located abundantly in the nuclei of *β*-cells in WTs. In TGs, insulin-positive cells were far fewer than in WTs. Even in the remaining *β*-cells of TGs, KPNA2 expression was observed (a typical cell is denoted with a yellow triangle).

To characterize the distribution of KPNA2 in the islets in greater detail, we conducted double immunostaining experiments with the combination of insulin and KPNA2 in mice of the late middle age group (Figure 4). In line with the results of the aged group of mice (Figure 3(d)), KPNA2 was highly expressed in the nuclei of pancreatic *β*-cells of WTs (Figure 4). Although some remaining *β*-cells still expressed KPNA2 to a considerable degree, the expression of KPNA2 in the *β*-cells was lower overall in *β*-cells of TGs (Figure 4).

**Figure 5:**
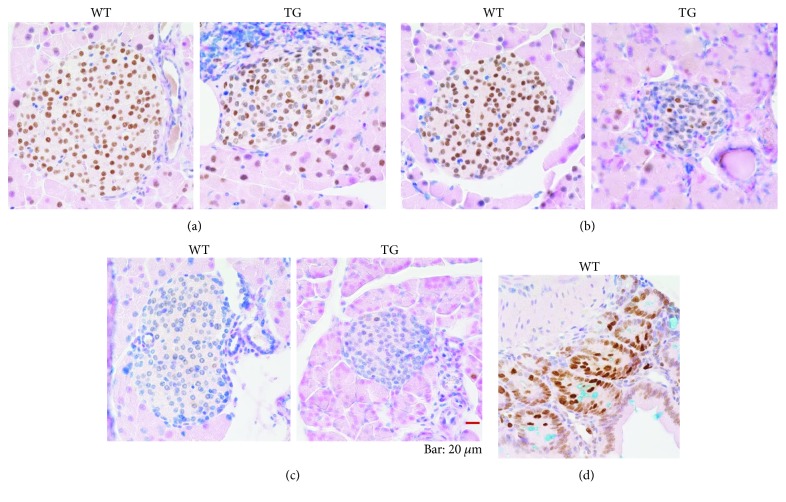
Immunostaining of islets for KPNA2 (brown) with alcian blue staining of WTs (left panels) and TGs (right panels) of aged (a), late middle-aged (b), and 4-week-old mice (c). As a positive control of mice at 4 weeks of age, the section of the small intestine from WTs was stained with antibodies to KPNA2 and with alcian blue staining (d). Bar, 20 *μ*m. Typical cases of islets are shown. At 4 weeks, the expression of KPNA2 in each cell in the islet was lower than that of mature ones. Bar, 20 *μ*m. Even at 4 weeks, as to the small intestine, strong immunostaining of KPNA2 in the cell nuclei (brown) was observed.

As for the age dependence, we compared the young mice (4-week-old) with the aged and late middle-aged ones. The expression level of KPNA2 in the nucleus of cells in islets at 4 weeks as a whole was remarkably lower than that of the mature stage (Figure 5). In the small intestine, distinct staining of KPNA2 was observed even in 4-week-old mice (Figure 5: the case of WTs is shown). These results indicate that the expression levels of KPNA2 in islets are quite low in comparison with those in mature stages. Our results also suggest that the expression levels of KPNA2 in the pancreatic endocrine cells are regulated in the developmental stage-dependent way and that KPNA2 plays some roles in the maturation of endocrine cells in the islet. To examine whether the difference in the expression level of KPNA2 exists between TGs and WTs at 4 weeks, we further conducted additional immunostaining experiments with KPNA2 only in mice of 4 weeks of age (Figure 1(a)). At 4 weeks, no remarkable difference between TGs and WTs in the staining level of KPNA2 was observed (Figure 1(a)), suggesting that there is no defect of KPNA2 expression in TGs at 4 weeks. As a characteristic feature, the variation of the level of KPNA2 among cell nuclei within the islet in the young mice was evidently greater than that in the adult WTs (Figure 1(a)). We also examined the levels of KPNA2 mRNA by real-time PCR using isolated islets derived from mice at 4 weeks. Consistent with the results of immunostaining (Figure 1(a)), there is no significant difference between two genetic groups in the mRNA level (Figure 1(b)). These results clearly indicate that TGs still maintain the same level of KPNA2 as that of WTs at the young stage. Taken together, these results suggest that the reduction of KPNA2 in Tg mice is responsible in some way for the dysfunction of *β* cells specifically in mature stages.

**Figure 6:**
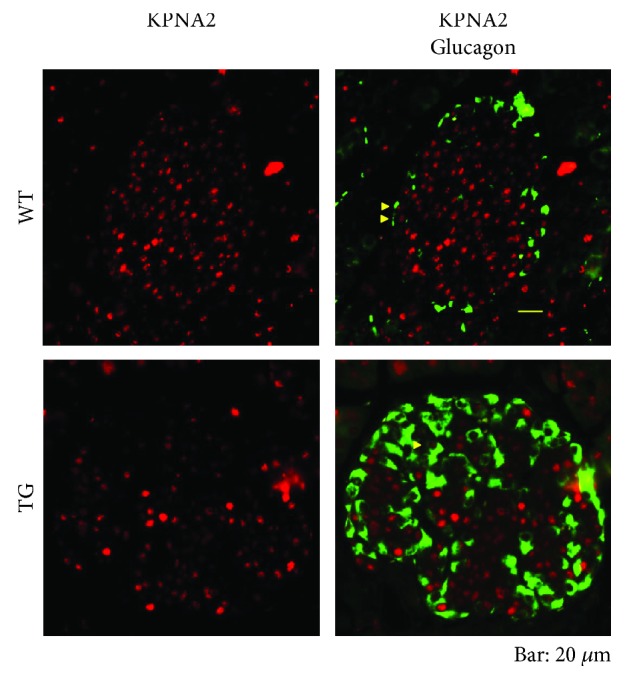
Coimmunostaining of islets for glucagon and KPNA2. Pancreas sections from aged group of mice [WTs (upper panels) and TGs (lower panels)] were costained with antibodies to glucagon (green) and KPNA2 (red) and stained with DAPI (blue) for nuclear staining. Bar, 20 *μ*m. As a whole in the islet, the expression of KPNA2 was much lower in TGs than in WTs. Typical cells with KPNA2 and glucagon double-positive are denoted by yellow triangles in both WTs and TGs. A noticeable tendency exists among cells in the islet: those cells weakly stained with KPNA2, colocalized more in glucagon-positive cells than in non-glucagon-positive cells (upper panels), which suggests that the expression of KPNA2 in *α*-cells is lower than that in *β*-cells. This tendency for KPNA2 expression is discernible also in TGs (lower panels). In TGs, glucagon-positive cells were observed throughout the islets.

We also conducted double immunostaining experiments for glucagon along with KPNA2 in mice of the aged group (Figure 6). In line with the results shown in Supplementary Figure 6, in TGs, pancreatic *α*-cells were distributed abnormally over the islet (Figure 6). In *α*-cells, KPNA2 expression was observed in both WTs and TGs (Figure 6). We further perceived a clear tendency among nuclei that those with weakly stained KPNA2 resided more in *α*-cells than in *β*-cells (Figure 6). Taken together, our results suggest that KPNA2 is more highly expressed in *β*-cells than in *α*-cells in the islet. KPNA2 was also located abundantly in the nuclei of acini cells (Figure 2(b)). The expression of KPNA2 in acinar cells was further confirmed by double immunostaining for amylase (an acinar marker) and KPNA2 (Supplementary Figure 7).

### 3.4. Fibrosis and Infiltration of Immune Cells in the Islet

**Figure 7:**
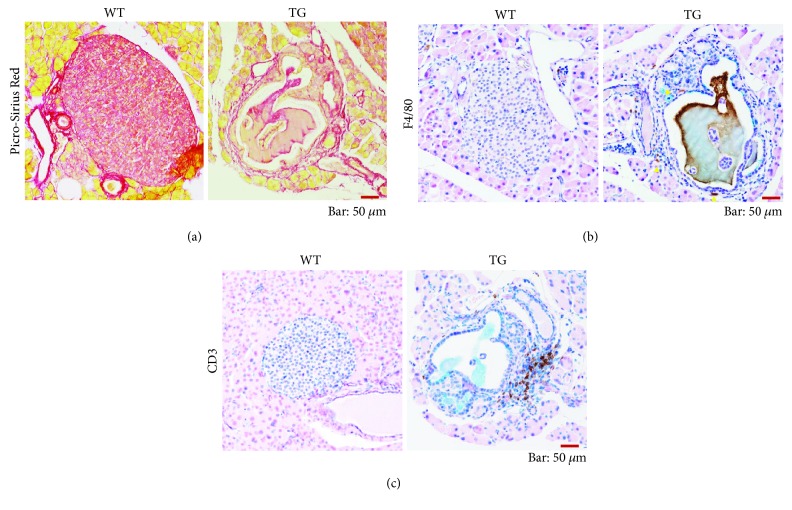
Islet fibrosis and infiltration of immune cells in the islets of aged group of mice (left panels, WTs; right panels, TGs). Representative cases are shown in each genetic group (in TGs, the islets harboring intra-islet ducts are shown). Bar, 50 *μ*m. (a) Picro-Sirius red staining of the islets. The fibrosis, shown in the red fibrotic structures, is discernible inside the islet of TG. The lumen in the intra-islet duct is also stained with Picro-Sirius red. In the WT case, almost no such structures were observed inside the islet. (b) Immunostaining of islets for F4/80 with alcian blue staining. Representative images are shown in each genetic group. No F4/80-positive cells are observed in the islet of WT. In contrast, several F4/80-positive cells (brown) are discernible near or inside the islets in TG (yellow triangles). The inner side of the component of lumen in the intra-islet duct is stained nonspecifically. (c) Immunostaining of islets for CD3 with alcian blue staining. CD3-positive cells (brown) are recruited to the islets of TG. No CD3-positive cells are observed in the islets of WTs.

The extent of fibrosis in the islet in aged TGs was assessed using Picro-Sirius red staining. Extensive fibrosis formation was clearly observed predominantly in islets of TGs (Figure 7(a)). At 4 weeks and 19 weeks, fibrosis was not discernible in both WTs and TGs (data not shown), indicating that the islet fibrosis progress with age. In aged TGs, the extent of the accumulation of leukocytes adjacent to islets was evidently more apparent than that of WTs (Supplementary Figure 6). Therefore, the types of subsets of immune cells adjacent to islets in aged TGs were examined using immunostaining against F4/80 (macrophages) and CD3 (T cells). In WTs, macrophages and T cells were only rarely observed adjacent to islets (Figure 7(c)). By contrast, in a considerable number of islets of TGs, the accumulation of large amounts of CD3-positive cells near and inside the islets was observed in TGs (Figure 7(c)). Smaller amounts of F4/80-positive cells were recruited to the islets in TGs (Figure 7(b)). These results suggest that the main leukocytes recruiting to islets in TGs are T cells. Almost no discernible presence of activated pancreatic stellate cells (PSCs) in the islets of WT and TGs was observed (data not shown), suggesting that activated PSCs play no major role for islet fibrosis in TGs.

### 3.5. Expression of Regenerating Genes (Reg Genes) in the Whole Pancreas

**Figure 8:**
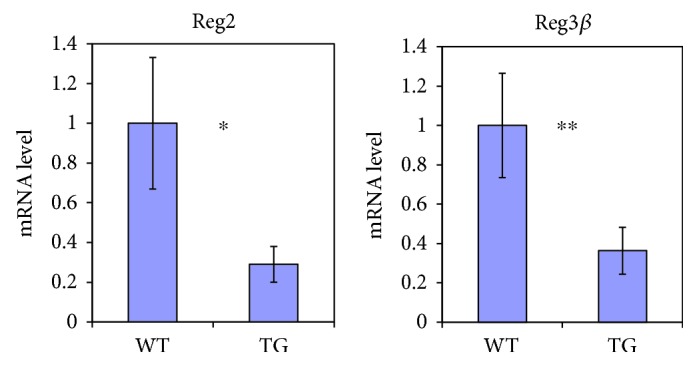
mRNA expression of Reg2 and Reg3*β* in the pancreas. Pancreases were collected from TGs as well as WTs at 4 weeks of age. The relative levels of mRNA were measured by real-time PCR and normalized to the corresponding mHPRT mRNA levels. In each representation, the mean value for WTs was set to 1. Data are means ± SE (WTs, *n* = 7; TGs, *n* = 6; ^∗^ *P* < 0.01 and ^∗∗^ *P* < 0.05, *t* test).

We also examined the expression of Reg2 and Reg3*β* genes in the whole pancreas of 4-week-old TGs by real-time PCR. The results demonstrated that the expression of Reg2 and Reg3*β* in TGs was significantly lower than that of WTs (Figure 8).

## 4. Discussion

Our results suggest that KPNA2 plays some important roles in the maintenance of the *β*-cell function because its expression is quite high in *β*-cells (Figure 4). Our results strongly suggest that, in the young stage (4 weeks of age), there is no defect of KPNA2 expression in TGs (Figure 1). By contrast, in late middle age and aged stages, the expression of KPNA2 protein in pancreatic *β* cells in TGs was clearly lower than in WTs (Figures 3 and 4). Some unknown functions of KPNA2 in *β* cells may remain in addition to its reported regulation of GLUT2 [17]. Taken together, the KPNA2 reduction is expected to contribute to the dysfunction of *β* cells in TGs at mature stages. Previously, we have demonstrated that, at 4 weeks, the level of blood glucose is still normal in TGs compared with that of WTs [5]. Although exact mechanisms of inducing the reduction of KPNA2 in the islet of Tg mice in mature stages are currently unknown, the possible transcriptional and/or posttranscriptional decrease of KPNA2 in the islet of TGs might be associated with glucotoxicity due to the high blood glucose in mature TGs [5]. Differences of the level of KPNA2 expression between *α*-cells and *β*-cells were observed (Figures 4 and 6). Consistent with our data (Figures 4 and 6), it was shown recently that KPNA2 is more expressed in *β*-cells than that in *α*-cells by RNA sequencing analysis ([21] as supplemental data). The implications of the phenomenon remain to be elucidated.

We also demonstrated that KPNA2 expression increases depending on the stage of ductal atypia. We showed that PanIN-like cells highly express KPNA2, although low expression of KPNA2 was observed in normal ductal cells (Figure 2(d) and Supplementary Figure 4). It is noteworthy that KPNA2 expression was high in mucin-rich PDG cells (Figures 2(b) and 2(c)). Our results newly uncovered some molecular characteristics of PDG cells. KPNA2 may perform crucially important functions related to the generation and/or maintenance of the ductal dysplasia outside islets. We also showed that, in the small intestine, especially high expression of KPNA2 was observed in the cells of crypts (Figures 5(d) and Supplementary 3), suggesting that progenitor-like features of cells are correlated with the level of KPNA2. Supporting that idea, PDG cells reportedly express several of the genes specific to progenitor cells in the intestinal cryptos [12].

Using a mouse model that conditionally overexpresses a constitutively active form of Akt in Pdx1-expressing cells and lineage tracing approaches, the occurrence of transdifferentiation from *β* cell to ductal cell was demonstrated in vivo [22]. The morphological features of the intra-islet duct-like cells in our TGs closely resemble those of mice which have introduced KRAS oncogene with a mutation (KrasG12D) in pdx-1-expressing cells in the adult stage [23]. In addition, by introducing KrasG12D into insulin-expressing cells, it was demonstrated that endocrine cells have some ability to transdifferentiate to ductal cells in condition of chronic inflammation in vivo [23]. Taken together, judging from the cases described in reports of the relevant literature, it seems most likely that the overexpression of C414A-CRY1 transdifferentiates pancreatic endocrine cells to ductal cells. Although exact mechanisms of the emergence intra-ductal cells from nonductal cells in the islet are currently unknown, dedifferentiated *β*-cells [24] and/or putative pancreatic stem cells [23] might be the source of intraductal cells under stress conditions such as inflammation, as discussed in the literature [23].

Regenerating proteins (REGs) are induced during regeneration of pancreatic tissue after pancreatectomy and have been proposed as growth factors for pancreatic beta cells [25]. We examined the possibility that REGs have some relations to the pathogenesis in TGs. We focused REG2 and REG3*β*, because the proteins are known to be abundantly expressed in pancreatic acinar cells in the normal condition [26] and induced in response to various pathological conditions such as diabetes and pancreatitis in mice. As to protective effects of the REGs in the endocrine and exocrine cells in the pancreas against the generation of diabetes and acute pancreatitis, its roles in the pancreas are still not decisive in spite of extensive studies using gene-manipulated mice [27–29]. Our result suggests the possibility that the reduced expression of Reg2 and Reg3*β* genes in the pancreas might have some roles in accelerating the pathophysiological conditions of the pancreas in TGs (Figure 8). Our results also suggest that the overexpression of mutant CRY1 affects the expression levels of Reg2 and Reg3*β* in the pancreas (Figure 8).

Senescent cells are known to increase the expression of genes encoding a series of secreted proteins. This senescent phenotype of cells is designated as senescence-associated secretory phenotype (SASP) [30]. These secretory proteins include inflammatory chemokine/cytokines, chemoattractant for leukocytes, various growth factors, and tissue remodeling factors [30]. Our previous data demonstrated that islet cells of TGs show a pattern of gene expression resembling that of cells showing SASP [3]. Actually, T cells and macrophages located frequently near the islets (Figure 7) might be recruited by secretory factors from *ß*-cells. They seem to function in roles as stimulators in developing the intra-islet ductal structures in TGs (Figure 7). The recruited immune cells might also have supportive effects on the maintenance of remaining *ß*-cells in TGs, as discussed previously in reports of the relevant literature [31, 32].

Our results suggest that activated PSCs play no major role in forming fibrosis in the islet of TGs. Consequently, it is likely that the SASP-like phenotype of islets of TGs itself is a prime cause for the islet fibrosis, which progresses with age. Results suggest that the development of PanIN and PDG is associated with islet inflammation using a human-IAPP-transgenic (HIP) rat model [14]. Considering reports of the relevant literature and our own results, the SASP-like inflammatory feature of islets in TGs [3] probably execute crucially important functions in the development of ductal dysplasia not only inside islets but also outside islets in TGs.

## 5. Conclusion

The results presented herein suggest that, in TGs, the reduction of KPNA2 expression in the islet might contribute to anomalies of the islet function in mature stages. Furthermore, a possible correlation was found between KPNA2 expression and the generation of ductal metaplasia. We demonstrated with overexpressing C414A-CRY1 in mice that fibrosis of islets accompanying ductal metaplasia in islets and the attraction of immune cells near islets occurred age-dependently. Taken together, our results have revealed some new aspects of islet pathophysiology, providing clues to understanding the development of ductal metaplasia. Our results indicate that the upregulation of KPNA2 can be a useful barometer for pancreatic ductal cells with atypical features. The molecular importance of the increment of KPNA2 in the formation of potentially precancerous ductal lesions remains to be elucidated.

## Figures and Tables

**Table 1:** Frequency of emergence of islet with atypical intra-islet ducts in each age of mice.

Months of age	Genetic group	Mice with atypical intra-islet ducts	Positive individual ratio (%)
		Number of positive/total number	
4.4	WT	0/6	0
	Tg	1/6	17
12-13	WT	0/7	0
	Tg	6/9	67
17-23	WT	0/9	0
	Tg	9/9	100

## Data Availability

The data used to support the findings of this study are included within the supplementary information file.

## Supplementary Materials

Supplementary 1 Figure 1: KPNA2 expression in PanIN-like ducts and PDGs in aged mice. Representative images of alcian blue-stained pancreas of WT mouse (left) and TG mouse (right). Bar, 100 *μ*m.

Supplementary 2 Figure 2: immunostaining of pancreatic sections from aged mice [WTs (left panel) and TGs (right panel)] for *α*-SMA counterstaining with alcian blue. Bar, 50 *μ*m. Representative images are presented for the respective genetic groups. *α*-SMA-positive cells (shown in brown) were observed in the fibrotic area around mucin-producing atypical ductal structures in TGs (yellow triangles).

Supplementary 3 Figure 3: immunostaining of the small intestine for KPNA2 with alcian blue. The section of the small intestine from the aged WT mouse was stained with antibodies to KPNA2 and with alcian blue staining. Bar, 20 *μ*m. KPNA2-positive cells (brown) were located in the epithelium cells of intestinal villi. A particularly high expression of KPNA2 was observed in the cells of crypts (yellow triangles).

Supplementary 4 Figure 4: immunostaining of pancreatic ductal cells in aged mice for KPNA2 with hematoxylin counter-staining. Pancreas sections from WTs (left panels) and TGs (right panels) were stained with antibodies to KPNA2. Bar, 20 *μ*m. Representative KPNA2-staining images of the normal duct (left) and the PanIN-like duct (right) are shown in upper panels. Corresponding images for negative controls without primary antibody are shown too in lower panels. Considerably strong staining of KPNA2 (brown) was observed in PanIN-like ductal cells. Weak but significant staining of KPNA2 was observed in the nuclei of normal ductal cells. Strong nonspecific staining (brown) was also observed in the duct lumen of the normal duct, which is known to often happen with the ducts.

Supplementary 5 Figure 5: immunostaining of pancreatic ductal cells (A) and islet cells (B) in aged mice for PDX-1 with hematoxylin counter-staining. Pancreas sections from WTs (left panels) and TGs (right panels) were stained with antibodies to PDX-1. Bar, 20 *μ*m. (A) Representative images of the normal duct (left panel), PDG, and PanIN-like ducts (right panel) are shown. Strong staining of PDX-1 (brown) was observed in the nuclei of PDG and PanIN-like ducts. (B) PDX-1 was located abundantly in the nuclei of islet cells in WTs (left panel). In TG (right panel), fewer nuclei were PDX-1-positive than with WT (left panel).

Supplementary 6 Figure 6: immunostaining of pancreatic islets in aged group of mice [WTs (left panels) and TGs (right panels)] for insulin (A) and glucagon (B), with alcian blue. Bar, 50 *μ*m. In TGs, insulin-positive cells (brown) were far fewer than in WTs. In TGs, glucagon-positive cells (brown) were observed throughout the islets. In TG, the accumulation of the cells having characters of leucocytes adjacent to islets is discernible (yellow triangle).

Supplementary 7 Figure 7: coimmunostaining of islets for amylase and KPNA2. Pancreas sections from late middle age of WT were costained with antibodies to amylase (green) and KPNA2 (red) and were counterstained with DAPI (blue) for nuclear staining. Bar, 20 *μ*m. KPNA2 was observed in the nuclei of acinus cells (yellow triangles) in addition to the nuclei of endocrine cells in the islets.
