# Supplementary figures and images for: Karyopherin Alpha 2-Expressing Pancreatic Duct Glands and Intra-Islet Ducts in Aged Diabetic C414A-Mutant-CRY1 Transgenic Mice
Source: J Diabetes Res. 2019 Apr 24;2019:7234549. doi: 10.1155/2019/7234549 (PMC6507265; doi:10.1155/2019/7234549)

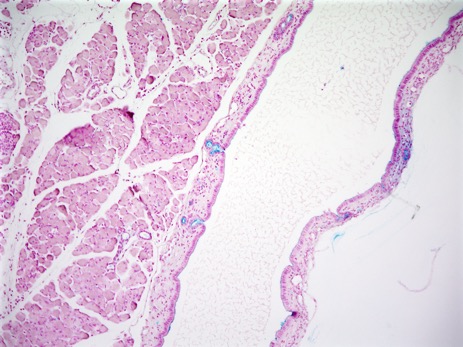

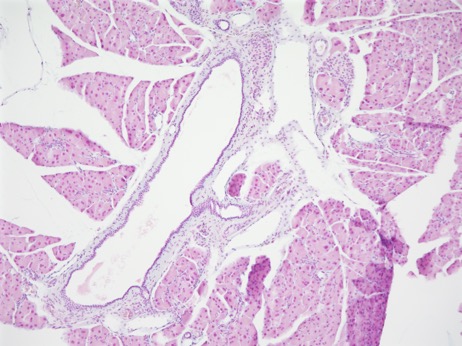


TG

WT

Bar: 100µm

Supplementary Fig 1

Supplement: Supplementary 1 — Figure 1: KPNA2 expression in PanIN-like ducts and PDGs in aged mice. Representative images of alcian blue-stained pancreas of WT mouse (left) and TG mouse (right). Bar, 100 μm. [file 7234549.f1.docx]

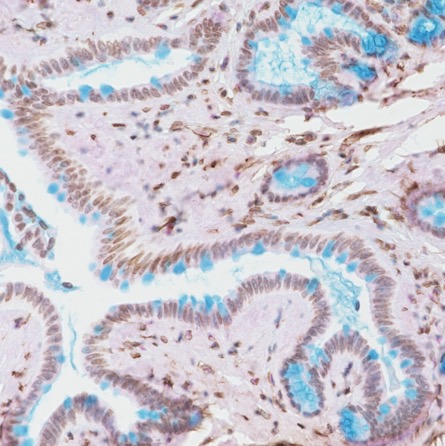

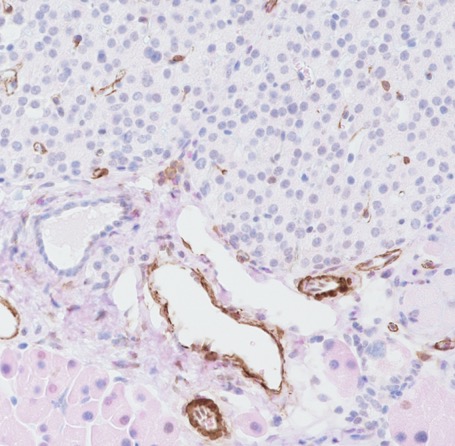


WT

TG

Supplementary Fig 2

islet

Bar: 50µm

Supplement: Supplementary 2 — Figure 2: immunostaining of pancreatic sections from aged mice [WTs (left panel) and TGs (right panel)] for α-SMA counterstaining with alcian blue. Bar, 50 μm. Representative images are presented for the respective genetic groups. α-SMA-positive cells (shown in brown) were observed in the fibrotic area around mucin-producing atypical ductal structures in TGs (yellow triangles). [file 7234549.f2.docx]

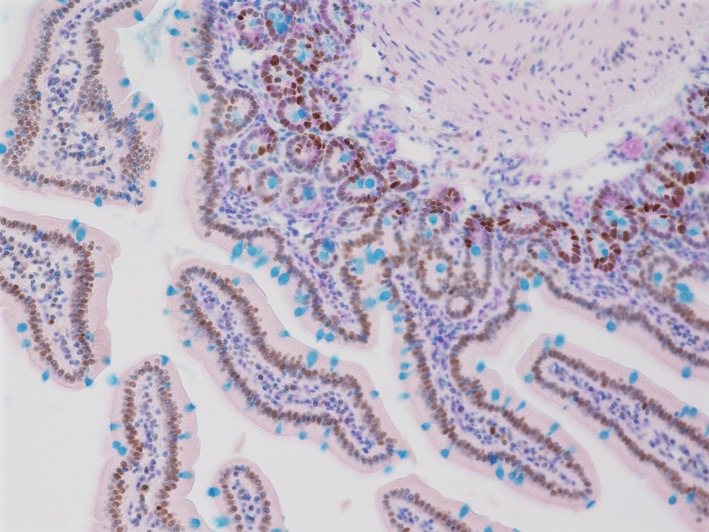


Supplementary Fig 3

Bar: 20µm

Supplement: Supplementary 3 — Figure 3: immunostaining of the small intestine for KPNA2 with alcian blue. The section of the small intestine from the aged WT mouse was stained with antibodies to KPNA2 and with alcian blue staining. Bar, 20 μm. KPNA2-positive cells (brown) were located in the epithelium cells of intestinal villi. A particularly high expression of KPNA2 was observed in the cells of crypts (yellow triangles). [file 7234549.f3.docx]

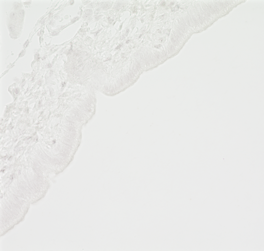

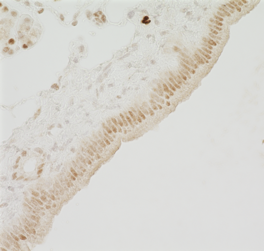

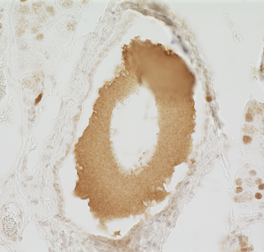

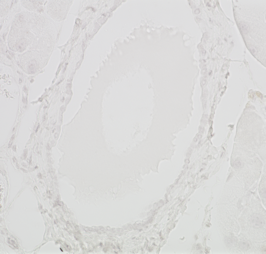


WT

TG

Supplementary Fig 4

KPNA2

Control

Bar: 20µm

lumen

Normal ductal

cells

lumen

PanIN-like cells

Supplement: Supplementary 4 — Figure 4: immunostaining of pancreatic ductal cells in aged mice for KPNA2 with hematoxylin counter-staining. Pancreas sections from WTs (left panels) and TGs (right panels) were stained with antibodies to KPNA2. Bar, 20 μm. Representative KPNA2-staining images of the normal duct (left) and the PanIN-like duct (right) are shown in upper panels. Corresponding images for negative controls without primary antibody are shown too in lower panels. Considerably strong staining of KPNA2 (brown) was observed in PanIN-like ductal cells. Weak but significant staining of KPNA2 was observed in the nuclei of normal ductal cells. Strong nonspecific staining (brown) was also observed in the duct lumen of the normal duct, which is known to often happen with the ducts. [file 7234549.f4.docx]

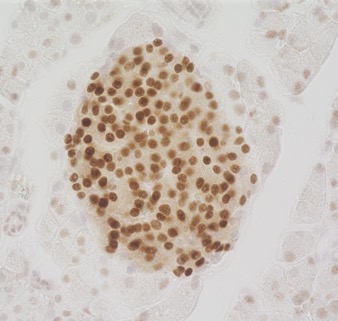

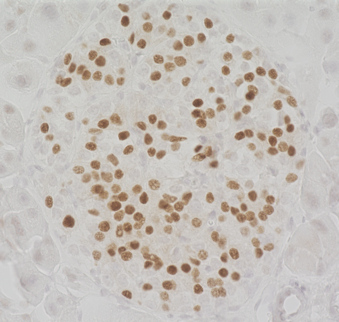

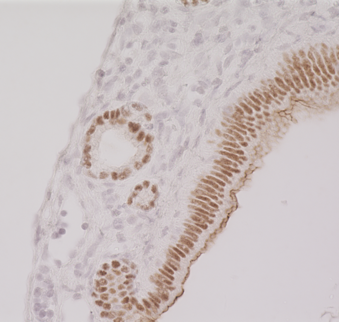

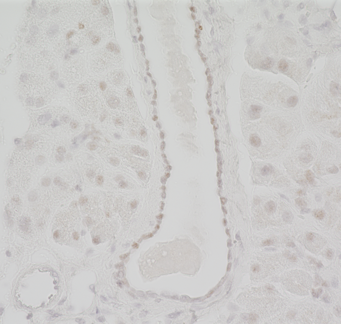


WT

TG

TG

WT

Supplementary Fig 5

(A)

(B)

Bar: 20µm

Supplement: Supplementary 5 — Figure 5: immunostaining of pancreatic ductal cells (A) and islet cells (B) in aged mice for PDX-1 with hematoxylin counter-staining. Pancreas sections from WTs (left panels) and TGs (right panels) were stained with antibodies to PDX-1. Bar, 20 μm. (A) Representative images of the normal duct (left panel), PDG, and PanIN-like ducts (right panel) are shown. Strong staining of PDX-1 (brown) was observed in the nuclei of PDG and PanIN-like ducts. (B) PDX-1 was located abundantly in the nuclei of islet cells in WTs (left panel). In TG (right panel), fewer nuclei were PDX-1-positive than with WT (left panel). [file 7234549.f5.docx]

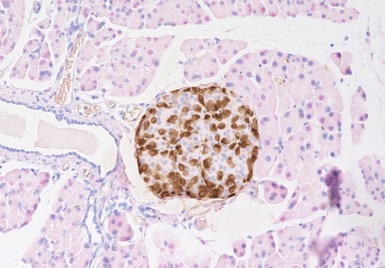

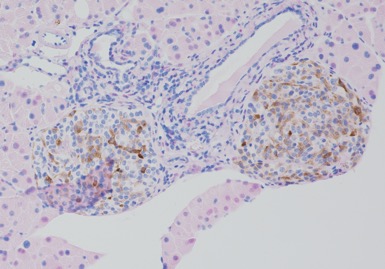

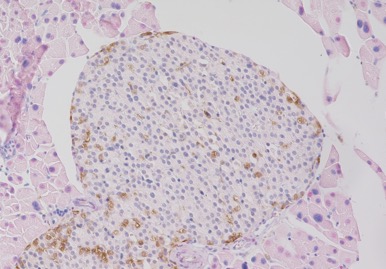

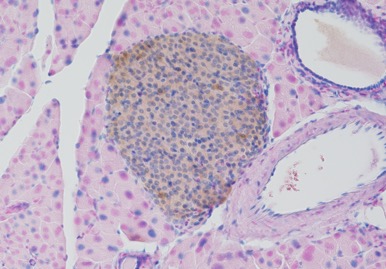


WT

TG

Supplementary Fig 6

(A)

(B)

Insulin

Glucagon

Bar: 50µm

Supplement: Supplementary 6 — Figure 6: immunostaining of pancreatic islets in aged group of mice [WTs (left panels) and TGs (right panels)] for insulin (A) and glucagon (B), with alcian blue. Bar, 50 μm. In TGs, insulin-positive cells (brown) were far fewer than in WTs. In TGs, glucagon-positive cells (brown) were observed throughout the islets. In TG, the accumulation of the cells having characters of leucocytes adjacent to islets is discernible (yellow triangle). [file 7234549.f6.docx]

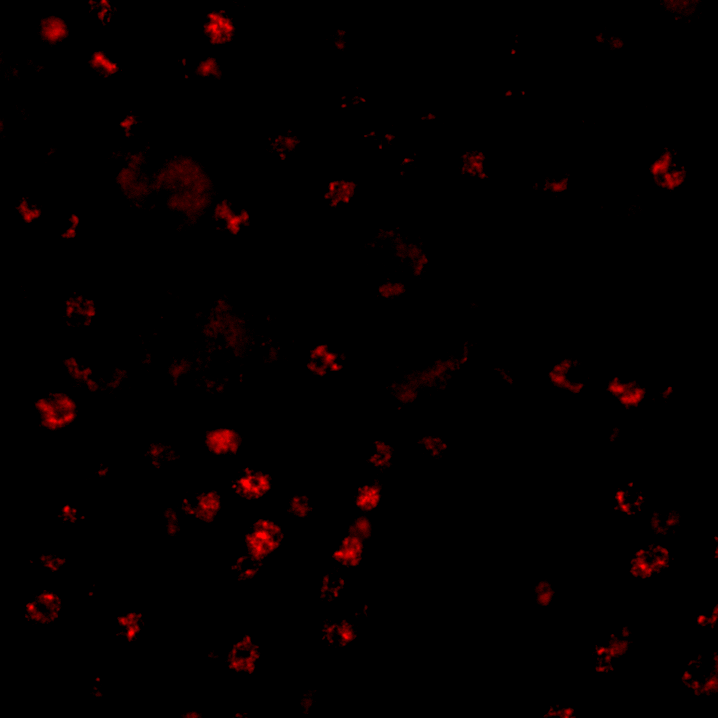

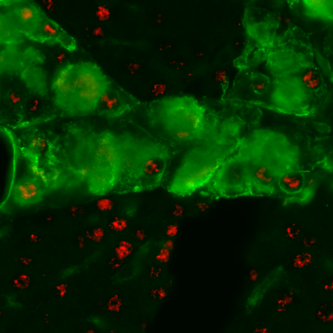

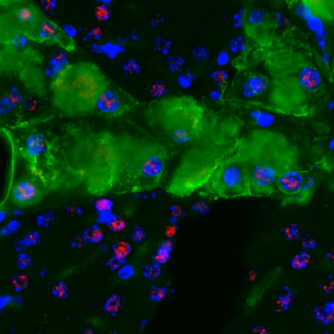


Supplementary Fig.7

B

A

C

islet

islet

KPNA2

KPNA2

amylase

KPNA2

amylase

DAPI

Bar: 20µm

Supplement: Supplementary 7 — Figure 7: coimmunostaining of islets for amylase and KPNA2. Pancreas sections from late middle age of WT were costained with antibodies to amylase (green) and KPNA2 (red) and were counterstained with DAPI (blue) for nuclear staining. Bar, 20 μm. KPNA2 was observed in the nuclei of acinus cells (yellow triangles) in addition to the nuclei of endocrine cells in the islets. [file 7234549.f7.docx]
